# Identification of Transcription Factor-Related Gene Signature and Risk Score Model for Colon Adenocarcinoma

**DOI:** 10.3389/fgene.2021.709133

**Published:** 2021-09-17

**Authors:** Jianwei Lin, Zichao Cao, Dingye Yu, Wei Cai

**Affiliations:** Department of General Surgery, Ruijin Hospital, Shanghai Jiao Tong University School of Medicine, Shanghai, China

**Keywords:** transcription factors, colon adenocarcinoma, risk score, bioinformatics, nomogram

## Abstract

The prognosis of colon adenocarcinoma (COAD) remains poor. However, the specific and sensitive biomarkers for diagnosis and prognosis of COAD are absent. Transcription factors (TFs) are involved in many biological processes in cells. As the molecule of the signal pathway of the terminal effectors, TFs play important roles in tumorigenesis and development. A growing body of research suggests that aberrant TFs contribute to the development of COAD, as well as to its clinicopathological features and prognosis. In consequence, a few studies have investigated the relationship between the TF-related risk model and the prognosis of COAD. Therefore, in this article, we hope to develop a prognostic risk model based on TFs to predict the prognosis of patients with COAD. The mRNA transcription data and corresponding clinical data were downloaded from TCGA and GEO. Then, 141 differentially expressed genes, validated by the GEPIA2 database, were identified by differential expression analysis between normal and tumor samples. Univariate, multivariate and Lasso Cox regression analysis were performed to identify seven prognostic genes (E2F3, ETS2, HLF, HSF4, KLF4, MEIS2, and TCF7L1). The Kaplan–Meier curve and the receiver operating characteristic curve (ROC, 1-year AUC: 0.723, 3-year AUC: 0.775, 5-year AUC: 0.786) showed that our model could be used to predict the prognosis of patients with COAD. Multivariate Cox analysis also reported that the risk model is an independent prognostic factor of COAD. The external cohort (GSE17536 and GSE39582) was used to validate our risk model, which indicated that our risk model may be a reliable predictive model for COAD patients. Finally, based on the model and the clinicopathological factors, we constructed a nomogram with a C-index of 0.802. In conclusion, we emphasize the clinical significance of TFs in COAD and construct a prognostic model of TFs, which could provide a novel and reliable model for the prognosis of COAD.

## Introduction

Colon cancer is one of the most common malignant tumors and the fifth leading cause of cancer-related death worldwide (Bray et al., 2018). Colon adenocarcinoma (COAD) is the most common pathological type of colon cancer (Fleming et al., 2012). Currently, the AJCC (American Joint Committee on Cancer) TNM staging system, together with age and gender, are the main prognostic indicators for COAD patients (Kim et al., 2015; Bruni et al., 2020). However, the significant differences in survival outcomes of COAD patients with the same clinicopathologic characteristics still exist, which signifies that a prognostic model based solely on clinicopathologic characteristics is of limited value (Nagtegaal et al., 2011; Bruni et al., 2020; Wang T. et al., 2020). Therefore, searching for highly specific and sensitive methods to predict the prognosis of COAD patients precisely is the key point of developing individualized treatment strategies for COAD patients.

Transcription factors (TFs) are vital cellular proteins for transcription of genes in human cells, which bind to target genes by recognizing promoters or enhancers of DNA sequences and affect many cellular functions, such as cell cycle and cell metabolism (Vaquerizas et al., 2009; Fitzgerald et al., 2014; Ehsani et al., 2016). As the terminal effectors of signaling pathways in cells, normal TFs play important roles in overall gene expression profiles (Xu H. et al., 2021). Recent studies have revealed the significance of deregulation TFs in COAD development and demonstrated that TFs associate closely with clinicopathological features and prognosis of COAD (Zhou et al., 2020; Xu H. et al., 2021). Meanwhile, several significant TFs, such as nuclear factor κB (NF-κB) and cAMP-response element-binding protein (CREB), have been found that aberrantly express in COAD and promote the development of COAD (Rayet & Gelinas, 1999; Hui et al., 2014). Hence, TFs may be important biomarkers for predicting prognosis, as well as potential targets for the treatment of COAD patients. However, recent studies mainly focused on the predicting value of TF-related genes; the present study constructed an original risk score model based on TF-related genes with remarkable predicting efficiency in COAD patients.

Though the efficacy of colon cancer treatment has improved in recent years with the advance in surgical methods and follow-up treatments, the mortality in patients with tumor infiltration *in situ* and distant metastasis remains high (Xu et al., 2020). In addition, overtreatment in patients may lead to adverse reactions related to chemotherapy and immunotherapy (Meyerhardt & Mayer, 2005). Therefore, there is an urgent need to build superior or comprehensive models to predict the overall survival (OS) of COAD patients to judge who are high-risk or low-risk patients so that the toxic harm associated with overtreatment is reduced and novel measures for treatment will be provided. In the present study, a risk model based on TF-related genes for COAD patients was constructed using data downloaded from The Cancer Genome Atlas (TCGA) database and validated in the Gene Expression Omnibus (GEO) database. By using multivariable Cox regression and survival analysis, we demonstrated that our risk model can be regarded as an independent prognostic factor of COAD. In general, our findings indicated that the TF-related risk model is of great predicting value in COAD patients and provides novel potential targets for COAD treatment.

## Methods

### Data Collection

The mRNA expression data (Workflow Type: HT Seq-FPKM) and clinical information pertaining to survival time for COAD patients downloaded from TCGA website (https://portal.gdc.cancer.gov/repository) were used as the training set. 437 samples (39 normal tissues, 398 COAD tissues), and 384 cases of COAD patients were collected. As well, data from the GEO (http://www.ncbi.nlm.nih.gov/geo/) database were used as the validation set. There are 177 and 579 COAD patients in the GSE17536 and GSE39582, respectively. The GEO samples were analyzed by Affymetrix Human Genome U133 Plus 2.0 Array platform. All cases from TCGA or GEO databases that miss the information were excluded from the analysis. The clinical characteristics of the patients (age, gender, stage, T stage, N stage, and M stage) were recorded. Unknown clinical characteristics were deleted. As for cases from TCGA, we removed one patient (TCGA-AZ-4323) whose risk score was significantly abnormal in our follow-up risk assessment.

### Identification of Differentially Expressed Genes and Prognostic Genes

Complete data of TF-related gene symbols were download from the Human Transcription Factor database (http://bioinfo.life.hust.edu.cn/HumanTFDB#!/). Differentially expressed genes were identified by the linear models for microarray data (limma) R package based on TCGA–COAD data. The Gene Expression Profiling Interactive Analysis (GEPIA2, http://gepia2.cancer-pku.cn/#general) database, which can analyze the difference of gene expression between tumor and normal tissue (Tang et al., 2017), was used to further screen for differentially expressed transcription factor-related genes. Cutoff values were set at a *p* value < 0.05 and |log2 fold change| >1. Differentially expressed genes related to the OS of patients were analyzed by univariable Cox analysis with “Survival” R package. A value of *p* < 0.05 was considered to have a statistically significant difference.

### Construction of Risk Score Model by Multivariate Cox and Lasso Regression

Multivariate Cox regression analysis with the “survival” R package and Lasso analysis with the “glmnet” R package were used to determine which genes can be used as predictive genes. The TF-related risk model was constructed by the result of multivariate Cox regression analysis, and each COAD patient risk score was calculated by the risk model to be divided into two groups (high- and low-risk subgroups) based on the median value. The risk score formula was as follows: $\;\text{Risk Score }=\;\sum_{i}^{7}\mathrm{Xi}*\mathrm{Yi}\;$ (X: coefficients, Y: gene expression level). Receiver operating characteristic (ROC) curves and Kaplan–Meier curves (K-M curves) were used to evaluate the ability of this risk model. Meanwhile, univariate and multivariate Cox regression analyses were also used to evaluate the prognostic efficiency of different clinicopathological features and our risk model.

### Alteration of Seven Genes in the Model and Protein–Protein Interaction Network

The cBioPortal dataset (https://www.cbioportal.org/), which contains genomic data from a variety of tumors, was used to study the genetic variability of transcription factor-related genes in our model. The STRING database (https://string-db.org/) is set for searching online for known protein interoperability relationships. We used this database to analyze and predict the functional relationship among TFs, and the cytoHubba plug-in in the software of Cytoscape (version3.8.1) was used to show the correlation between TFs in our risk model and other TFs.

### The Construction of a Nomogram to Estimate the Clinical Outcome of Colon Adenocarcinoma Patients

We used the “rms” R package to construct a nomogram. Subsequently, calibration curves were used to test the association between the predicted outcome and the actual situation in 1, 3, and 5 years. The GSE39582 was used as the validation dataset for the nomogram.

### Gene Set Enrichment Analysis

Gene set enrichment analysis (GSEA), as a computational method, can be used to detect significantly enriched and depleted group of genes (Subramanian et al., 2005). In this study, GSEA was regarded as an approach to explore potential molecular mechanisms underlying the expressions of TFs in our risk model. The group was divided into high-expression and low-expression groups according to the median value of each transcription factor expression level. Necessary files were finished and uploaded into the GSEA 4.1 software. The gene set database was “c2. cp.kegg.v7.2. symbols.gmt [Curated]” and “c5. go.v7.4. symbols.gmt [Gene ontology].” The number of permutations were conducted 1,000 times in the analysis. The phenotype labels were high and low. Normal *p*-value <0.05 were enriched.

### Correlation of the Genes and Risk Score With Clinicopathologic Features and Immune Cells

Clinical association analysis was used to assess the relationship between risk model and clinicopathologic features by using the “beeswarm” R package. Tumor immune microenvironment is crucial for the antitumor immunity in cancer, so we analyzed the relationship between our risk model and immune cell infiltration by getting the matrix of immune cells from the Tumor Immune Estimation Resource (TIMER) (Bhattacharya et al., 2018), which is a database that provides the systematic analysis of immune infiltrates in cancer. In this database, users can predict the abundance and proportion of six immune cell subsets (B cells, CD4^+^ T cells, CD8^+^ T cells, dendritic cells, macrophages, and neutrophils) in tumor samples. The relationship between the risk model and immune cell infiltration was constructed by the Pearson’s correlation test in the R software.

### Statistical Analysis

R version 4.0.5 and Perl version 5.28 were used to perform statistical analysis. Excel office 2019 was used to organize data from TCGA and GEO database. A value of *p* < 0.05 was regarded as significant.

## Results

### Overall Design of the Study

**FIGURE 1:**
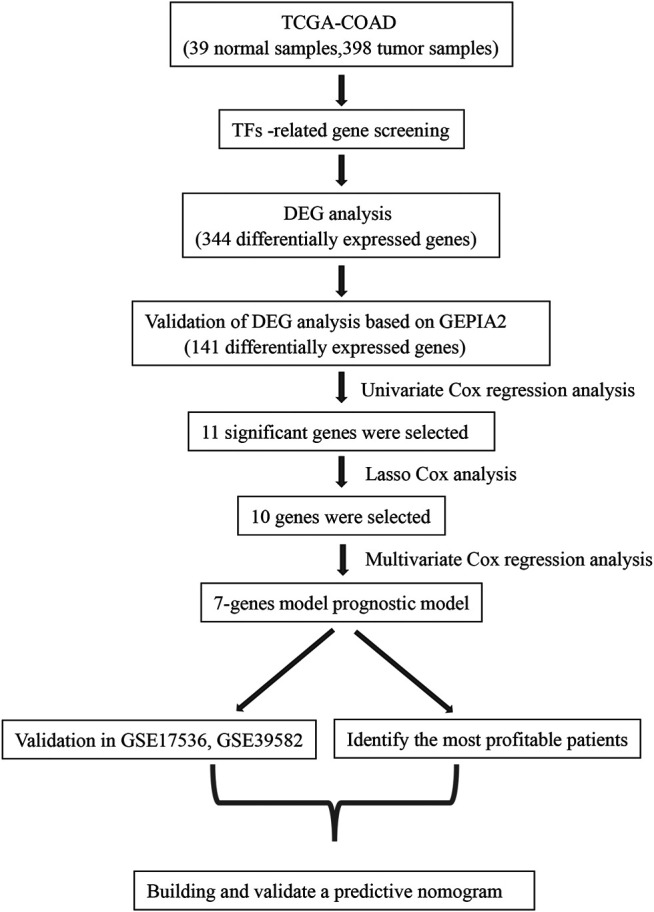
The workflow to construct the transcription factor (TF)-related risk model in COAD patients. TCGA, The Cancer Genome Atlas; COAD, colon adenocarcinoma; DEG, differentially expressed gene; GEPIA2, Gene Expression Profiling Interactive Analysis 2.

The flow chart for this study is shown in Figure 1. Gene expression data and clinical data of COAD patients were downloaded from TCGA database. Some of the clinical information of 384 patients from TCGA are shown in Table 1. According to the Human Transcription Factor database and differential expression analysis, 344 differentially expressed TF-related genes in patients with COAD were screened. Then, the GEPIA2 database was used to validate 344 differentially expressed genes, and 141 differentially expressed genes were screened finally. Lasso and multivariate Cox regression analysis were used to construct a prognostic risk model for patients with COAD. Then the K-M and ROC curves were used to assess the seven TF-related risk models. Two data sets of the GEO database were used as external cohort to validate the model. Finally, the nomogram of COAD was constructed by using TCGA data. Calibration and C-index were used to assess the nomogram.

**TABLE 1:** The clinical information of colon adenocarcinoma (COAD) patients from The Cancer Genome Atlas (TCGA).

	Patient *n* = 384	
Age	≤65	159
	>65	225
Gender	Female	180
	Male	204
Stage	Stages I–II	216
	Stages III–IV	157
	Unknown	11
T	T1–2	77
	T3–4	306
	Tis	1
M	M0	285
	M1	54
	Mx	39
	Unknown	6
N	N0	230
	N1–2	154

Note. T, T stage; N, N stage; M, M stage; Stage, TNM stage.

### Differential Gene Analysis

**FIGURE 2:**
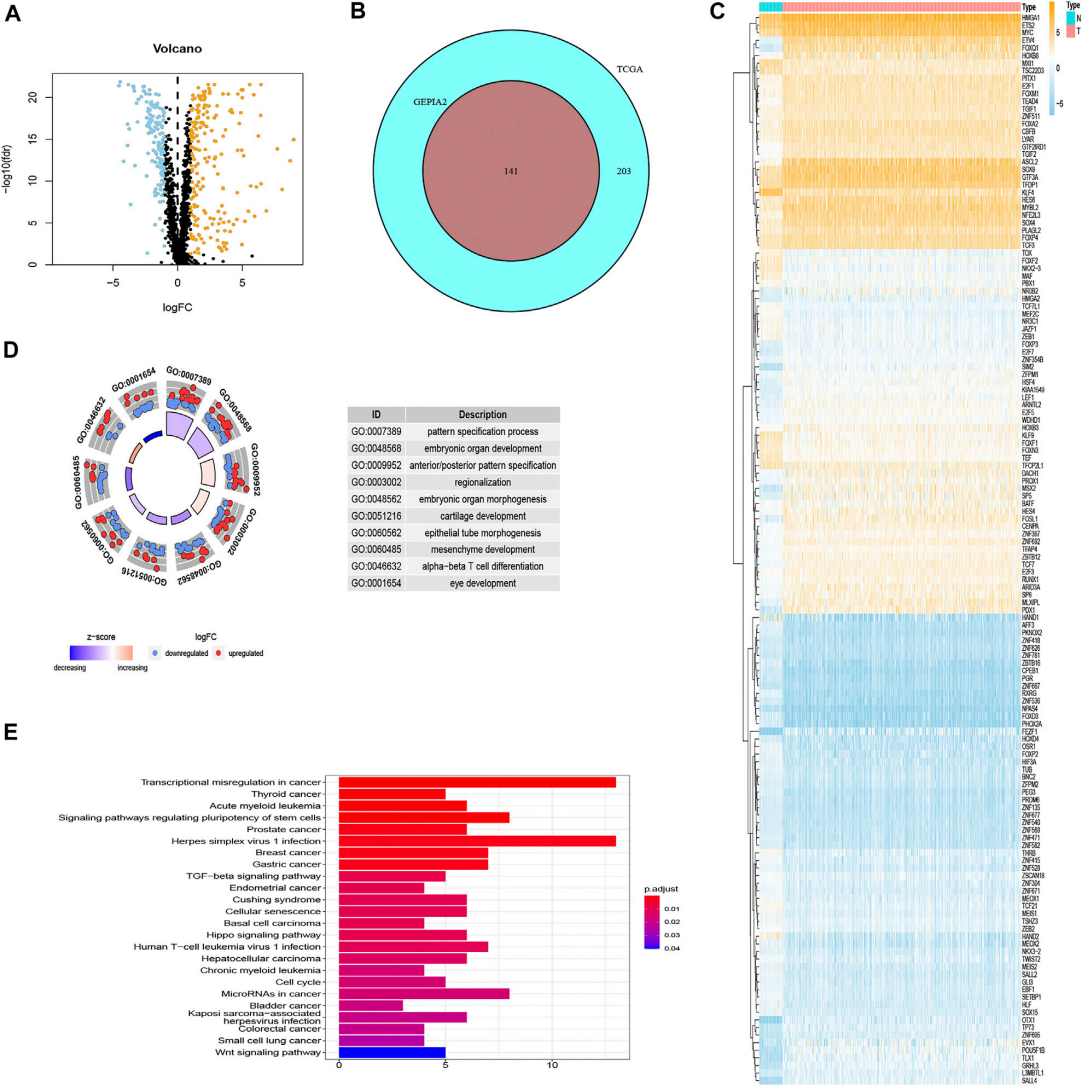
The results of differential gene analysis. **(A)** The volcano plot of differentially expressed TF-related genes based on TCGA. The sky blue points are the downregulated genes, the orange points are the upregulated genes, representing *p* < 0.05, |log_2_FC| > 1. **(B)** The Venn plot to show the same differentially expressed TF-related genes between TCGA and GEPIA2 database. **(C)** The heatmap of differentially expressed TF-related genes. The vertical axis refers to genes, the horizontal axis refers to differences in gene expression between tissues, the orange means high expression, and the sky blue means low expression. **(D)** Gene Ontology (GO) circle graph of the top 10 GO terms with the most enriched genes. **(E)** Bar graph of the top 24 Kyoto Encyclopedia of Genes and Genomes (KEGG) pathway with the most enriched genes; the vertical axis refers to names of pathways, and the horizontal axis refers to the number of genes.

Three hundred forty-four differentially expressed TF-related genes, which were analyzed from TCGA database, are shown in Figure 2A. Then, 141 differentially expressed TF-related genes were screened from 344 differentially expressed TF-related genes by using the GEPIA2 database, including 70 downregulated genes and 71 upregulated genes (Figure 2B). The expression information of 141 genes in COAD were also shown by a heatmap (Figure 2C) between normal and tumor tissues, which was more obvious. Gene Ontology (GO) analysis showed 141 genes were mainly concentrated on pattern specification process and embryonic organ development, and other significantly differentially expressed gene GO terms were exhibited in a circle graph (Figure 2D). In the Kyoto Encyclopedia of Genes and Genomes (KEGG) pathway enrichment analysis, the result is shown by a bar plot in Figure 2E. These results suggested that the genes screened in this study are related to tumorigenesis and the development pathway.

### Construction of Risk Score Model by Univariate, Multivariate, and Lasso Cox Regression Analysis

**FIGURE 3:**
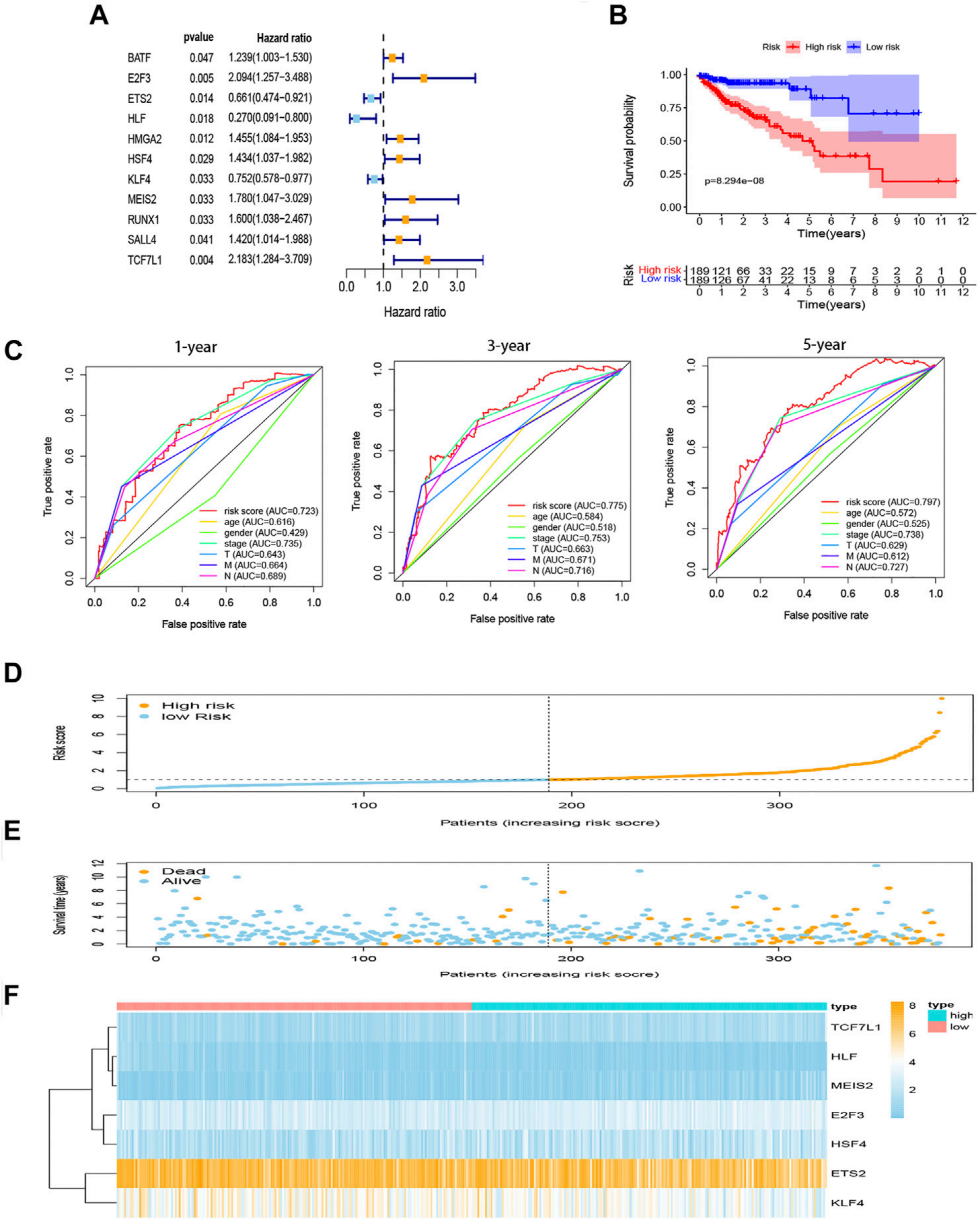
Construction of prognostic model for COAD. **(A)** Hazard ratio of univariate Cox analysis for DEGs. **(B)** Survival analysis to verify the prognostic model. **(C)** ROC curve to evaluate the predictive efficacy of the risk model. **(D)** Distribution of risk scores of each COAD patient. **(E)** Correlation between survival time and survival status of each patient. **(F)** The expression pattern of seven TF-related genes. DEGs, differentially expressed genes. ROC, receiver operating characteristic.

In TCGA set, 141 TF-related genes were considered in the univariable Cox regression analysis with a *p*-value <0.05 as the threshold to distinguish which genes were related to the prognosis of COAD patients (Figure 3A). There were 11 genes associated with the prognosis of COAD. Then, the Lasso regression (Supplementary Figures S1A,B) and multivariable Cox analysis (Table 2) were used to do further identification of TF-related genes associated with the prognosis of COAD patients, and the coefficient of each TF-related gene was calculated in order to obtain the risk score. After analyzing, seven genes were selected from 11 genes to construct a risk score model. The minimum Akaike information criterion (AIC) of the risk model was 683.96. Furthermore, the median value of the risk score from training and validation set was used as the cutoff value to divide patients into low-risk and high-risk groups.

**TABLE 2:** The results of multivariate Cox analysis in COAD.

ID	Coefficients	HR	HR.95L	HR.95H	*p*-Value
E2F3	0.735185	2.085867	1.1849	3.671907	0.010836
ETS2	−0.29493	0.744583	0.504,494	1.098931	0.137551
HLF	−2.29211	0.101,053	0.02909	0.351037	0.000309
HSF4	0.341165	1.406,585	0.970863	2.03786	0.071289
KLF4	−0.21757	0.804475	0.608,272	1.063964	0.127,187
MEIS2	0.91836	2.505179	1.296842	4.839387	0.006262
TCF7L1	0.551359	1.735609	0.954879	3.154683	0.070526

Note. HR, hazard ratio.

### Risk Score Model

Seven genes (E2F3, ETS2, HLF, HSF4, KLF4, MEIS2, and TCF7L1) were taken into our risk model. Based on the coefficient of each gene from multivariable Cox regression analysis, the risk score can be calculated by the formula shown in the method with the coefficients in Table 2.

The K-M curves show that the high-risk group performed with significantly poorer prognostic outcomes (Figure 3B) than the low-risk group. Multi-indicator ROC curves were plotted, and the AUCs of the risk model were as follows: 1-year AUC: 0.723, 3-year AUC: 0.775, 5-year AUC: 0.797 (Figure 3C). The risk score, survival time, and gene expression of the seven genes in every COAD patient are vividly shown in Figures 3D–F.

**FIGURE 4:**
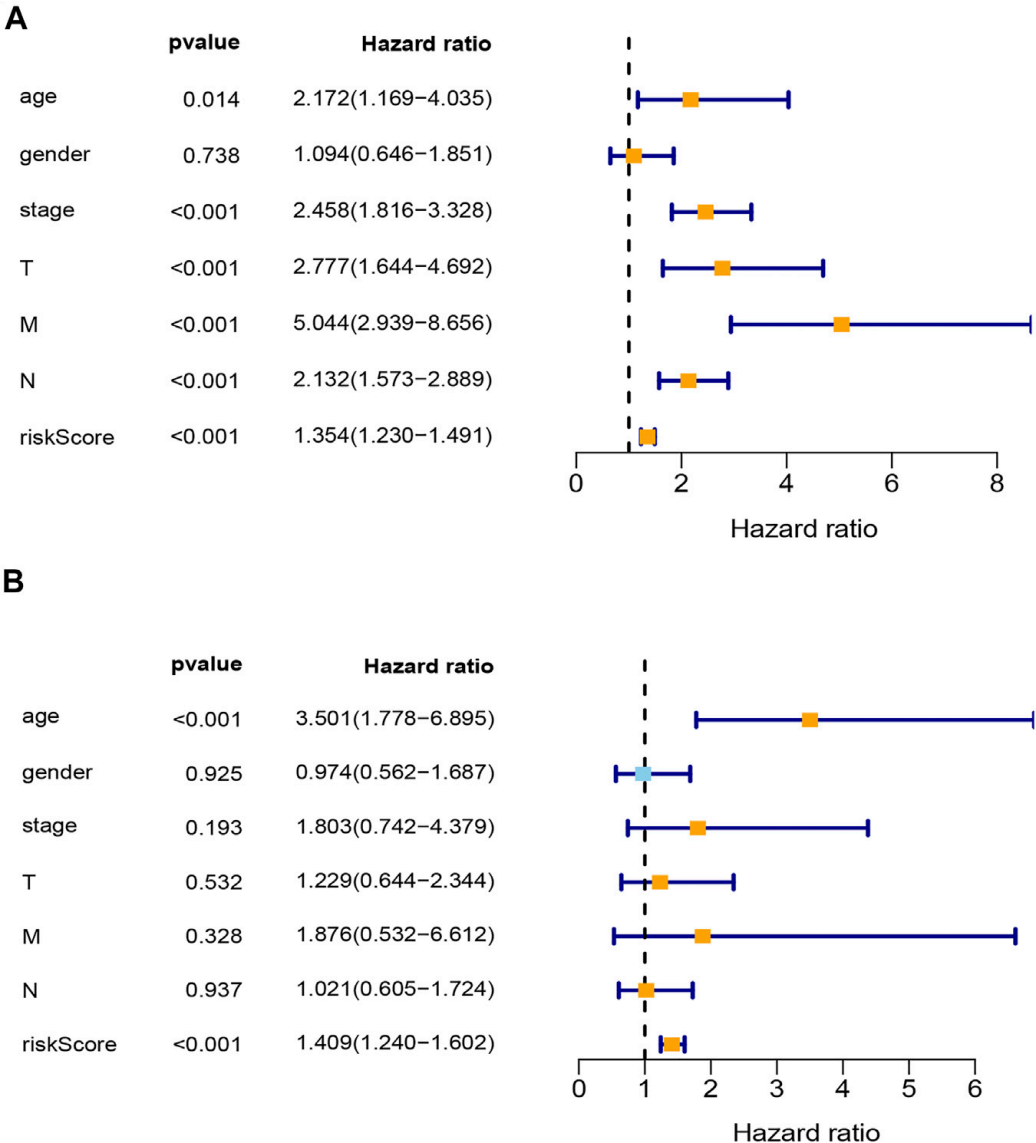
Analysis of clinical independence for riskScore. **(A)** Univariable Cox regression analysis for clinical characters and risk score. **(B)** Multivariable Cox regression analysis for clinical characters and risk score. T, T stage; N, N stage; M, M stage; stage, TNM stage; riskScore, risk score model.

Univariable and multivariable Cox analyses were used to identify whether risk score and clinicopathologic features (age, gender, AJCC stage, T stage, N stage, and M stage) could be the independent prognostic indicator. The results of univariable Cox analysis show that age, AJCC stage, T stage, N stage, M stage, and risk score were significantly correlated with OS (*p* < 0.05; Figure 4A). Meanwhile, the results of multivariable Cox analysis show that age and risk score were still significantly associated with OS (*p* < 0.05; Figure 4B). These results suggested that the risk score model could be the independent prognostic indicator for COAD.

To better understand COAD patients who can get more benefit from our risk score model, we divided these patients into several subgroups: age ≤65 and age >65, female and male, Stages I–II and Stages III–IV, T1–T2 and T3–T4, N0 and N1–2, and M0 and M1. In our K-M curves, age ≤65, male, Stages III–IV, T3–T4, N1–2, and M0 seem more suitable for our risk score model (Supplementary Figure S2A–L). These results suggest that our risk score model was strongly related to tumor progression.

### Validation of Transcription Factor-Related Risk Score Model in Gene Expression Omnibus Datasets

**FIGURE 5:**
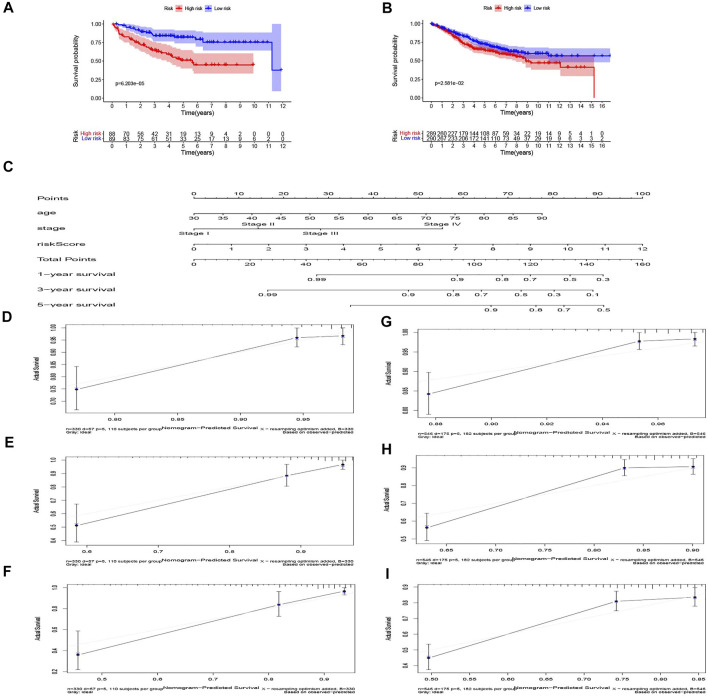
Validation of the risk model in the GEO dataset and nomogram for predicting survival rate in COAD patients. **(A)** GSE17536 and **(B)** GSE39582 dataset. **(C)** The nomogram for predicting the 1, 3, and 5-year survival rate by age, stage, riskScore. **(D–F)** The 1, 3, and 5-year calibration curves of TCGA dataset. **(G–I)** The 1, 3, and 5-year calibration curves of Gene Expression Omnibus (GEO) dataset.

In GSE17536, survival data from 177 patients were used for external validation of our risk model. The risk score of each patient was calculated using the coefficients from TCGA data, and the patients were divided into high-risk group and low-risk group according to the median of the risk score. K-M curve analysis demonstrated better survival outcome in the low-risk group (Figure 5A). Similarly, in GSE39582, 579 patients were used as external validation of our risk model. The results also showed significant differences in OS among patients at different risk groups in this dataset (Figure 5B).

### Genetic Alternation of Seven Transcription Factor-Related Genes and Protein–Protein Interaction Network

The cBioPortal database was used to analyze the mutations of seven genes, which showed that E2F3, ETS2, HLF, HSF4, KLF4, MEIS2, and TCF7L1 were altered in 8, 8, 6, 6, 5, 9, and 6% of 524 colorectal adenocarcinoma patients separately (Supplementary Figure S3A). As shown in Supplementary Figure S3B, genes were altered in 34.94% of 332 COAD patients and 36.03% of 136 rectal adenocarcinoma patients. The change in the expression of mRNA in cancer was the main change type for these genes. The PPI network based on the STRING database and Cytoscape software showed that KLF4, MEIS2, and TCF7L1 interacted more with other transcription factors (Supplementary Figure S3C). In total, our results suggest that the establishment of a transcriptional-related gene model makes up for the fact that a single transcriptional gene is not a good indicator of the condition of a patient.

### Construction and Validation of Nomogram Model

Age, TNM stage, and risk score of TCGA dataset were taken into consideration to establish the nomogram to forecast the survival probabilities of 1, 3, and 5-year overall survival time (Figure 5C). In our nomogram, each factor had its score according to their contribution in the risk of survival. The calibration curves were plotted to judge whether the actual survival time was in line with the predicted survival rate in 1, 3, and 5 years (Figures 5D–F). The C-index of this nomogram in predicting overall survival time was 0.802. The result of GSE39582, which was the validation dataset of the nomogram is shown in Figures 5G–I. These results show that the nomogram in our study can predict the actual survival outcome well.

### Gene Set Enrichment Analysis Revealed Seven Transcription Factor-Related Gene Signaling Pathways

GSEA was used to detect the potential signaling pathways and gene function of seven TF-related genes in COAD between low- and high-expression datasets. The signaling pathways and gene function, of which the nominal *p*-value <0.05 in the enrichment of “c2. cp.kegg.v7.2. symbols.gmt [Curated]” and “c5. go.v7.4. symbols.gmt [Gene ontology],” were taken into consideration. The results are shown in Supplementary Figures S4A–G, which suggested that most of genes were associated with tumors, and their abnormal expression may influence the development of related tumors. These genes were also involved in some tumor-related signaling pathways, such as TGF beta, P53, and MAPK. In addition, the genes in the model also can affect the signal pathways of apoptosis, immunity, and metabolism. As for GO analysis, the results analyzed by GSEA show that the seven genes may participate in cell metabolism and cell adhesion. In total, the GSEA results suggested that our risk model may participate in tumor-related, immune and metabolic signaling pathway, and related genes can affect cell growth and adhesion.

### Correlation of the Risk Score With Clinicopathologic Features and Immune Cells

**FIGURE 6:**
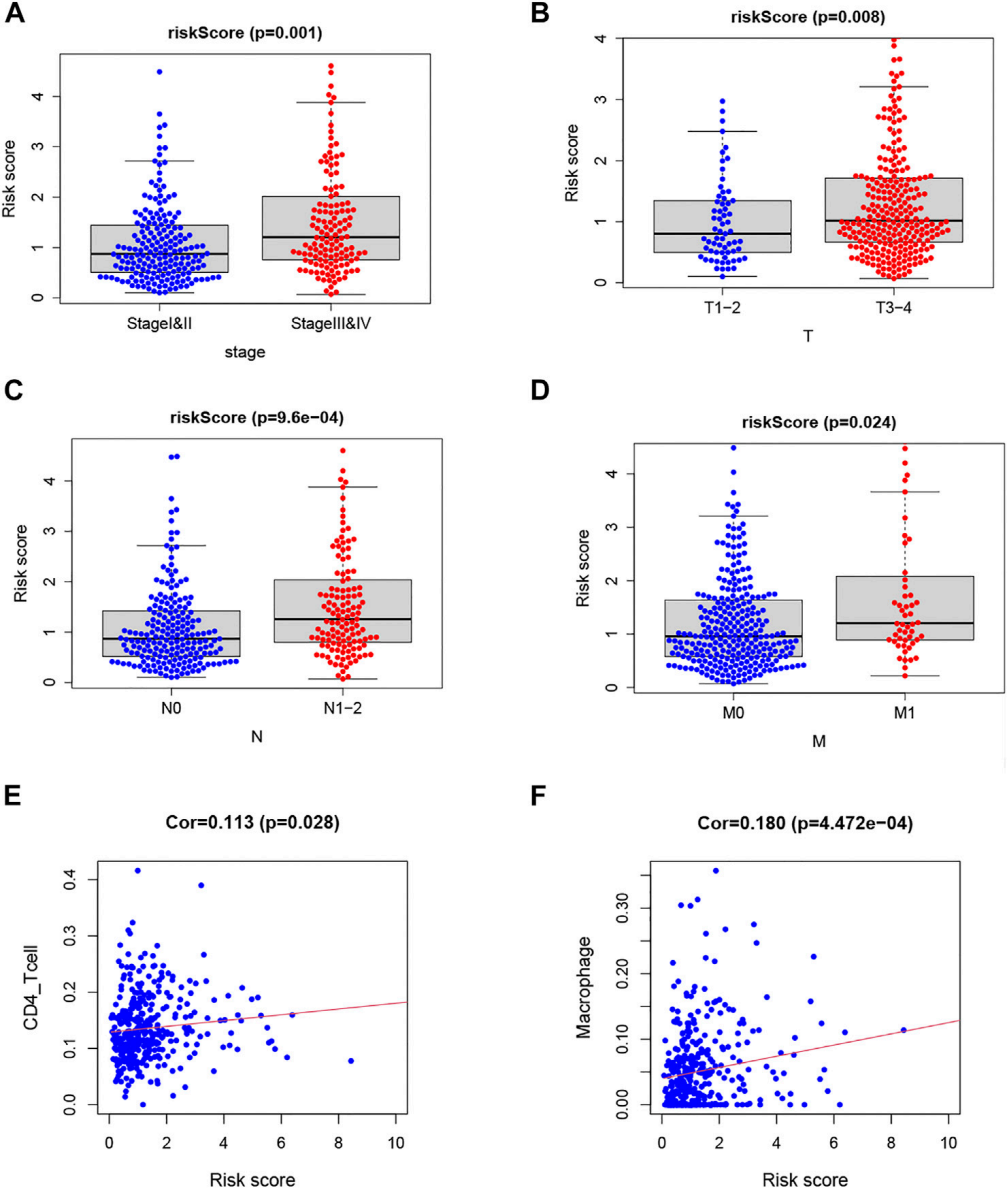
Analysis of clinical and immunological relevance for riskScore. **(A–D)** Analysis of relationship between clinicopathological factors and riskScore. **(E,F)** Analysis of relationship between immune cells and riskScore.

The risk score was significantly correlated with the clinicopathological features of TCGA-COAD (*p* < 0.05; Figures 6A–D). High risk score is associated with Stages III–IV, T3–4, N1–2, and M1, which meant that the TF-related risk model can reflect the progression of the COAD. Meanwhile, because the immune microenvironment is important for the tumors, we also analyzed the correlation between risk score and immune cells, which is shown in Figures 6E,F. The results showed that the risk score was related to the CD4 T cell and macrophage. These results suggested that the risk score based on TFs may reflect the tumor progression and the infiltration of immune cells in tumor microenvironment.

## Discussion

The present study indicated that the prognostic model based on TFs can predict the prognosis of COAD well. Meanwhile, the correlation between risk score and clinicopathologic factors as well as immune cells showed that the model was associated with poor clinicopathologic factors and immune cells. In GSEA analysis, seven TFs were found to be involved in signaling pathways associated with tumor development.

TFs are important components of cells that participate in the transcription of genes and many other biological processes (Dynlacht, 1997; Accili & Arden, 2004). About one-third of human developmental disorders are associated with abnormal TFs (Boyadjiev & Jabs, 2000). Many studies have investigated the role of TFs in tumors and the corresponding mechanisms. Dong et al. found that the transcription factor can support the development of breast cancer (Dong et al., 2020). Zhan et al. reported that transcription factors play a key role in the development of hepatoblastoma (Zhan & Zhao, 2020). Meanwhile, studies have also reported the role of TFs in the development of COAD (Rayet & Gelinas, 1999; Hui et al., 2014). However, the study of prognostic transcription factors is still lacking. As the terminal effector molecule of the cell signal pathway, TFs play important roles in the development of tumor, which makes it very important to study their function in predicting the prognosis of patients. The bioinformatics was used as a research tool to analyze data from a database to build and validate a prognostic model and to analyze its association with clinical features and immunity. In our study, data from TCGA were used as training set, and data from GEO were used as validation set to construct a seven-TF-related risk model by using multivariable Cox and Lasso regression. The prognostic model can predict the prognosis of patients well, which will be helpful in clinical evaluation and provide new therapeutic targets.

Seven TF-related genes were identified in this study, which have been reported separately regarding their roles in tumor. E2F transcription factors (E2F3), which can interact with histone acetyltransferase and induce cells to enter the cell cycle, participated in the development and progression of many tumors, such as pancreatic cancer and ovarian cancer (Kurtyka et al., 2014; Rotgers et al., 2014; Park et al., 2015; Pengcheng et al., 2021; Xu D. et al., 2021). In COAD, E2F3 played a key role in carcinogenesis by promoting the expression of cyclin D1 and CDK2 (B. Yang et al., 2020). V-ets erythroblastosis virus E26 oncogene homolog 2 (ETS2) can act as both a tumor suppressor and an oncogene (X. Ma et al., 2019). In COAD, ETS2 can induce oxaliplatin resistance and promote the malignant behavior of tumor cells (Wang H. et al., 2020). Hepatic leukemia factor (HLF), a kind of leukemia zipper transcription factor, can regulate circadian rhythms (Inaba et al., 1994). A study of hepatocellular carcinoma suggested that the role of HLF in inducing sorafenib resistance and in promoting tumor progression by activating c-Jun may help to discover new targets for cancer therapy (Xiang et al., 2019). Heat shock factor 4 (HSF4) can regulate cellular proliferation and differentiation (Jin et al., 2012). In cancer research, HSF4 can promote EMT in liver cancer cells to stimulate cell proliferation and invasion and was associated with poor prognosis in COAD patients (P. Ma et al., 2020; Y. Yang et al., 2017). Krüppel-like factor 4 (KLF4) can inhibit the development of tumor by associating with the non-Warburg metabolic behaviors (Blum et al., 2021). Low expression of KLF4 may lead to poor prognosis and link to the progression of COAD (Taracha-Wisniewska et al., 2020). T-cell factor 7-like 1 (TCF7L1) has been found to have a high expression in many cancers, such as breast cancer and skin squamous cell carcinoma (Slyper et al., 2012; Ku et al., 2017). It is the suppressor of the Wnt target gene expression and cell circle is the method for it to promote the development of COAD (Murphy et al., 2016; Eshelman et al., 2017). These studies indicate that seven transcription factors are associated with tumor. Our risk model can be well applied to evaluate the prognosis of patients with COAD.

Survival analysis based on clinical feature groups showed that our risk model was better able to predict the prognosis of young men with COAD at the advanced TNM stage. In addition, our risk model associated with advanced TNM stage and immune cells (CD4 T cell and macrophage). These results indicated that our risk model is related to the tumor microenvironment and affects the prognosis of patients with COAD (Diederichsen et al., 2003; Ye et al., 2019).

The present study, similar to another study using TF-related genes to construct a model of COAD, reveals the role of TFs in predicting the prognosis of COAD (Liu et al., 2020). However, when validated with the GEPIA2 database, the expression of TF-related genes in another article does not show the difference between normal and tumor samples. In contrast, the TF-related genes used in our article showed significant differences between normal and tumor samples in the validation of the GEPIA2 database. At the same time, our study goes further to combine clinical factors to construct a nomogram in order to improve the prognostic ability in COAD. A study that constructed a prognostic model based on immune-related genes reveal that TFs may regulate the expression of immune-related genes (Sun et al., 2020). By combining the results in our study, it was shown that TFs may participate in the tumor immune microenvironment. However, the model based on immune-related genes show that ROC was only 0.719, which was lower than that of our model. Therefore, our study showed that the genes in our model have significant difference between normal and tumor tissues, and our risk score model shows better ability in predicting the prognosis of COAD patients.

Though our model has shown remarkable ability in predicting prognosis of COAD patients, there are still a few limitations. This prognostic model still requires data of patients from other large cohorts to validate and not just take advantage of the data on the network database. Some of the TF-related genes in our model, which are not studied for mechanisms in COAD, still need to be revealed in our future studies.

## Conclusion

We identified seven TF-related genes, including E2F3, ETS2, HLF, HSF4, KLF4, MEIS2, and TCF7L1, and constructed a risk score model, which can predict the prognosis of COAD well. Moreover, these genes were associated with the prognosis of COAD patients and related to the development of cancer. Therefore, we thought our finding could help distinguish the COAD patients in the clinic, and the seven TF-related genes can become biological targets to treat COAD patients.

## Data Availability

The original contributions presented in the study are included in the article/Supplementary Material, further inquiries can be directed to the corresponding author.
