# Supplementary material for: Identification of Transcription Factor-Related Gene Signature and Risk Score Model for Colon Adenocarcinoma
Source: Front Genet. 2021 Sep 17;12:709133. doi: 10.3389/fgene.2021.709133 (PMC8485095; doi:10.3389/fgene.2021.709133)
Supplement: Supplementary file 1 [file DataSheet1.docx]

**Supplementary Information for**

**Identification of Transcription Factor-Related Gene Signature and Risk Score Model for Colon Adenocarcinoma**

Jianwei Lin^1†^, Zichao Cao^1†^, Dingye Yu^1†^, Wei Cai^1*^

^1^Department of General Surgery, Ruijin Hospital, Shanghai Jiao Tong University School of Medicine. Shanghai, Shanghai, China

* Correspondence:

Wei Cai

[caiwei@shsmu.edu.cn](mailto:caiwei@shsmu.edu.cn)

This file includes:

Figure S1-S4

**Supplementary Figure S1**

**
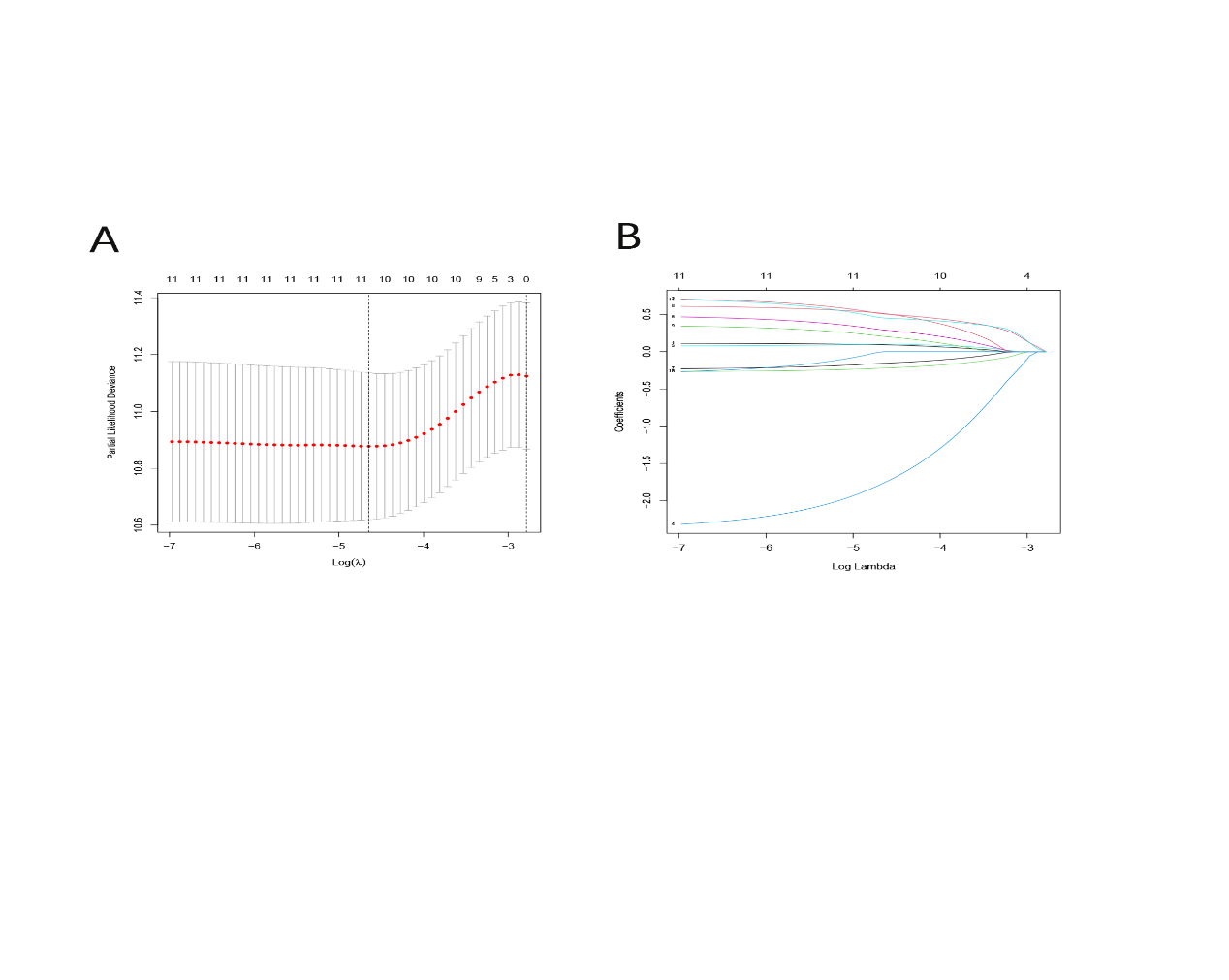
**

**Supplementary Figure S1.** Genes in prognostic model analyzed by Lasso Cox regression. (A) Distribution of Lasso analysis for eleven genes. Two vertical lines represent lambda.min and lambda.lse; (B) Coefficients for eleven genes analyzed by Lasso.

**Supplementary Figure S2**

**
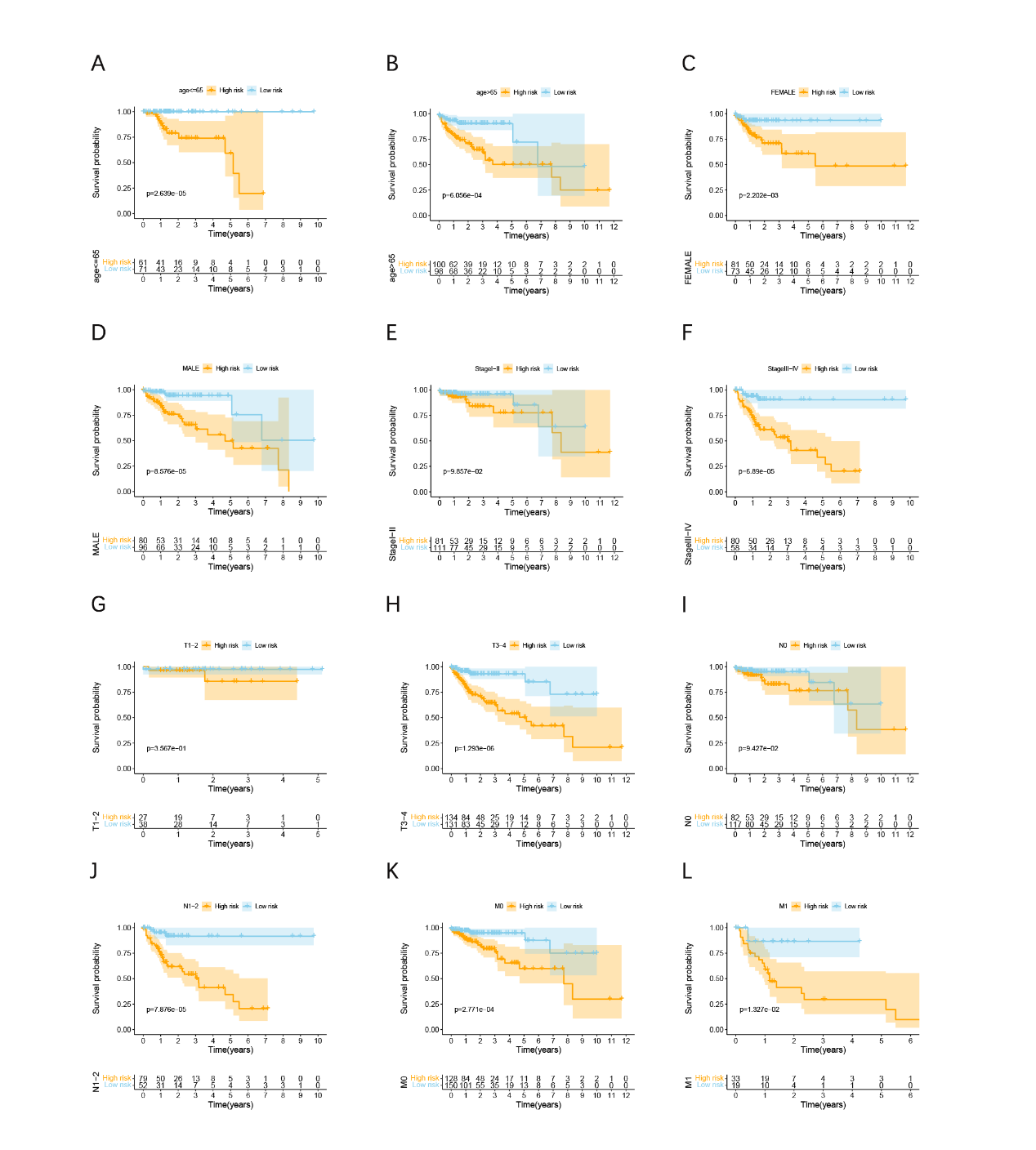
**

**Supplementary Figure S2.** Identify COAD patients suitable for risk model. (A–L) Survival analysis for risk score model in different patients. (A, B) Grouped by age. (C, D) Grouped by gender. (E, F) Stage I-Stage II is divided into one group, Stage III-Stage IV into another group. (G, H) T1-T2 is divided into one group, T3-T4 into another group. (I, J) M0 is divided into one group, M1 into another group. (K, L) N0 is divided into one group, N1-2 into another group.

**Supplementary Figure S3**

**
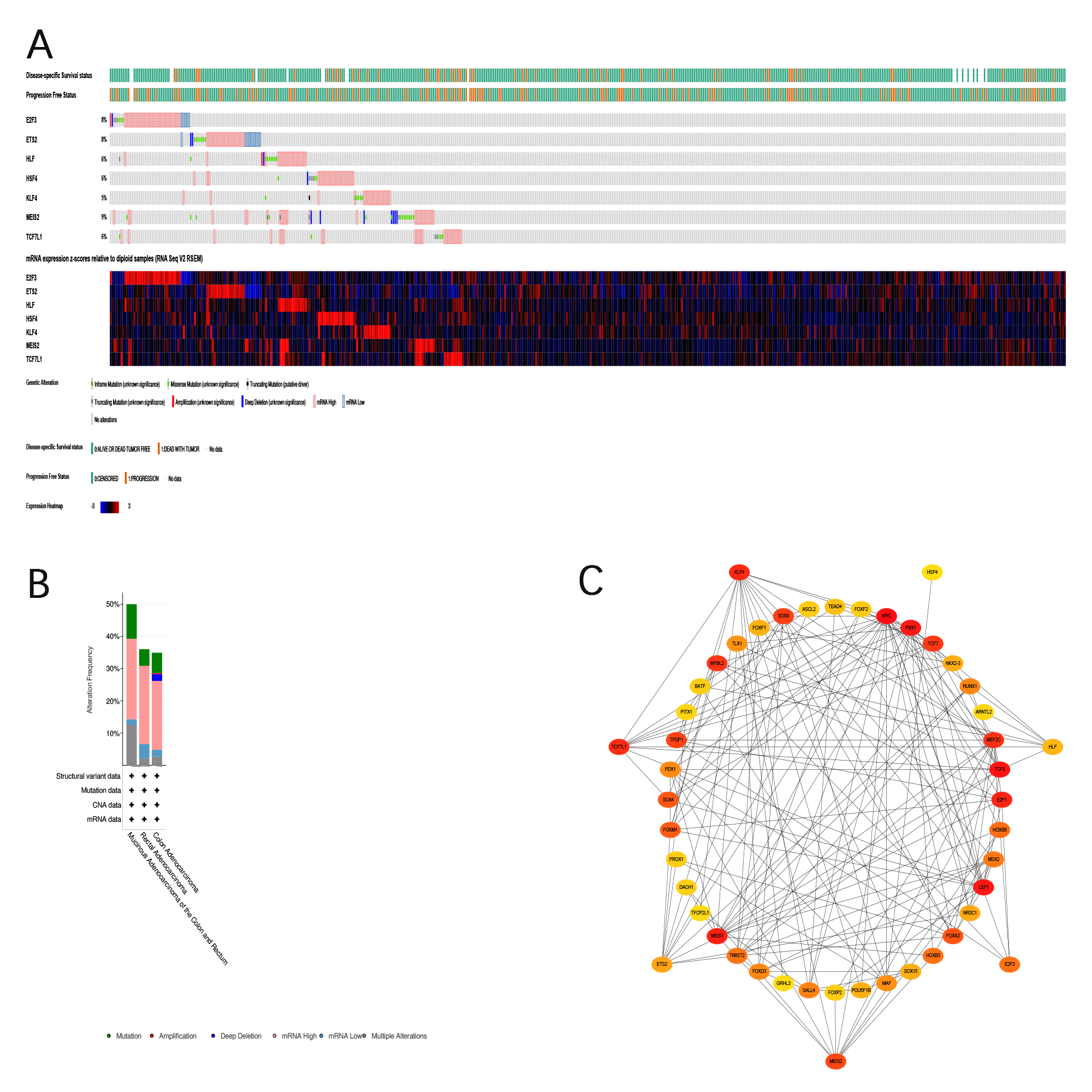
**

**Supplementary Figure S3.** Genetically alternation of seven TFs-related genes and PPI network. (A) A visual summary of gene alternation from colorectal cancer. (B) The total alternation of seven genes. (C) MCC degree made by Cytoscape screen genes. The deeper the red color, the higher the degree.

**Supplementary Figure S4**

**
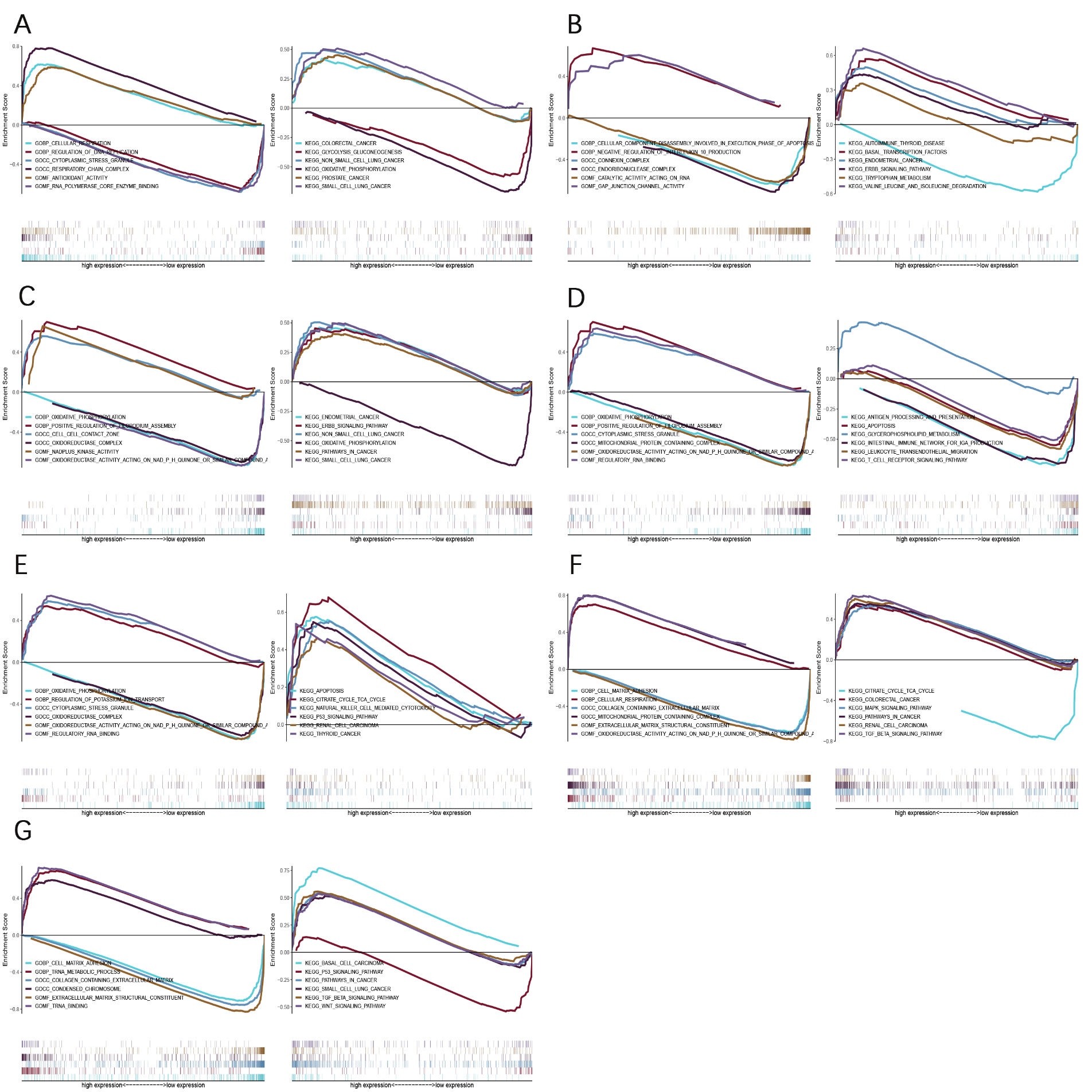
**

**Supplementary Figure S4.** GSEA analysis for seven genes in GO and KEGG. (A) GSEA analysis for E2F3; (B) Analysis for ETS2; (C) Analysis for HLF; (D) Analysis for HSF4; (E) Analysis for KLF4; (F) Analysis for MEIS2; (G) Analysis for TCF7L1. GSEA, Gene Set Enrichment Analysis. KEGG, Kyoto Encyclopedia of Genes and Genomes. GO, Gene Ontology.
